# Review of “Bioinformatics: A Computing Perspective” edited by Shuba Gopal, Anne Haake, Rhys Price Jones and Paul Tymann

**DOI:** 10.1186/1748-7188-4-9

**Published:** 2009-06-24

**Authors:** Dae-Won Kim, Hong-Seog Park

**Affiliations:** 1 Genome Research Center, Korea Research Institute of Bioscience and Biotechnology, Daejeon 305-806, Korea

## Overview

The passion of scientists in interdisciplinary fields to understand living beings gave rise to the field of bioinformatics. These scientists employ a number of methodologies and tools to deal with massive amounts of biological data. 'Bioinformatics: A Computing Perspective' is a comprehensive compilation of biological basics, computational methods, and modern approaches for resolving biological problems well suited to individuals with background education in computer science. In addition, this book provides long-running examples and discussions throughout the text to help students understand complex biological ideas.

## Contents

This book consists of nine chapters. The first chapter provides a history of scientific works and a definition of bioinformatics. Topics include the organization and roles of a bioinformatics team and the challenges of computational algorithms in molecular biology. Chapter 2 illustrates some overall basics of biology, including cellular organization and complexity, evolution, and how the language of cells is encoded at various steps through DNA, RNA and protein. This chapter also provides a description of the management of genetic data, including replication and verification of DNA. Chapter 3 deals with fundamental wet lab techniques, describing the principles of hybridization, cDNA synthesis, DNA sequencing methods and proteomics techniques. This chapter emphasizes the importance of development of appropriate computational approaches to working with biological data to provide an understanding and characterization of those data.

Chapters 4 and 5 provide a broad overview of fragment assembly, aimed especially at answering two main questions: What is the problem of biological sequence assembly? and What are the various computational approaches used for sequence alignment? Chapter 4 addresses the issue of the nature of genome sequences. It provides the reader with a basic example and simple pattern matching and graph algorithms for solving the sample problem. Chapter 5 provides descriptions of more extensive and advanced alignment algorithms for more exact similarity detection, such as Deterministic finite-state automata (DFAs), the Needleman-Wunsch algorithm, and the Smith-Waterman algorithm. This chapter also deals with evolutionary and heuristic approaches, including point accepted mutation (PAM).

Chapter 6 introduces the concept of evolution and computational methods for the discovery of evolutionary relationships based on the tree of life. This chapter offers a detailed introduction regarding how best to build phylogenies, including discussion of neighbor joining, maximum likelihood and maximum parsimony approaches. This chapter may be of particular interest to biological scientists who conduct evolutionary studies, since the models discussed in this chapter are commonly used in analysis of the prehistory of an organism.

Chapters 7 and 8 provide a discussion of the principle of decipherment of genome sequences. Chapter 7 focuses on approaches for analyzing genomic sequences to identify biologically meaningful patterns. This chapter provides mathematical methods for assessing consensus sequences using position-specific weight matrices (PSWM) and finding gene structure and open reading frames (ORF) via Hidden Markov Models (HMM). Chapter 8 briefly introduces molecular mechanisms and provides a flood of biological data. Further, it deals with the analysis of microarray expression data for understanding complicated phenomena in biology. This chapter provides a description of various array-based technologies and a discussion of the procedures and the computational approaches to the analysis of gene expression data, including gene filtering, scaling, normalization and transformation. In addition, it includes worked examples and a discussion of clustering algorithms (Hierarchical clustering, K-means clustering, self-organizing map) and classification methods (genetic algorithms, support vector machines).

The final chapter touches on several different issues, many of which are cutting edge in the field of bioinformatics. It provides a brief but lucid treatment of the visualization of complex biological datasets, non-coding RNA function prediction, drug discovery through protein structure, the mapping of complex diseases, and systems biology.

## Summary

Bioinformatics is a vast multidisciplinary field. *Bioinformatics: A Computing Perspective *does a good job of compiling the relevant topics about the state of the art and challenges facing bioinformatics in the field of biology. This book has several strong points. Although it is consisting of many chapters and subtitles dealing with detailed computing techniques and experimental methodologies, a diversity of attempts have been made to bring together descriptions of bioinformatics, biological basics, detailed experimental methodologies and computing algorithm for understanding biological complexes. The style is very readable, and discusses both the biology and the computation of every topic presented. In particular, this text places emphasis on practical skills involved in analyzing genes and proteins, extracting sequence data, and identifying genes and proteins implicated in disease by providing ample illustrative examples and extensive java codes. Many algorithms are built up in steps, showing how successive insights from both computation and biology can make existing techniques work better. Consequently, this book is inspiring readers to design the breakthrough computational approaches and algorithms that will provide novel and valuable insights into biological phenomena.

In summary, this book provides an excellent resource for those initiating a study of biology based on computer science and provides interesting facts to give life to lectures on this topic. Most of the topics discussed show a fascinating snapshot of current progress in bioinformatics using advanced technologies. Moreover, the examples in this book help provide an understanding of the complicated algorithms described.

## Competing interests

The authors declare that they have no competing interests.

## Authors' contributions

DK: conception, drafting and final approval of the manuscript. HP: drafting and final approval of the manuscript.

